# Derangements of the Thyroid Gland

**Published:** 1901-12-07

**Authors:** 


### DERANGEMENTS OF THE THYROID GLAND.

The effects produced by the removal of the thyroid gland are so marked, and the results of treating myxedema by thyroid extract have been so surprising, that it is not unnatural that its use in other conditions in which no such striking results have been obtained should have led to a general feeling that the therapeutic uses of thyroid extract have but a narrow range; one limited, in fact, to the relief of such symptoms as are known to be producible by the removal of the gland. Still, one meets here and there with cases in which this extract is decidedly useful, and anyone who can so group them and give such a clinical picture of them as shall guide us to the sort of case in which we may expect benefit from the administration of thyroid extract does us a good service. Hence much interest attaches to a paper read by Dr. John Perry[1] at the October meeting of the Harvard Medical Society of New York on the "Functional Disorders of the Thyroid Gland." In this paper he shows that a certain group of cases which have been confused with rheumatism are in some way connected with disorder of the thyroid and are capable of being relieved by the administration of thyroid extract. In the cases described five symptoms were usually present, of which pain in the joints was the most prominent. The others were a feeble and rapid heart; a tendency to headache, not localised but general; menorrhagia, in women; and usually the fact that the development of the symptoms came on after a shock or fright. An important point was that most of the cases observed had in consequence of the joint pain been regarded as cases of rheumatism or gout. The joints were not red and usually were not very tender, but there was frequently a pallid swelling with slight tenderness, resembling the condition which develops in the so-called neural arthropathy described by Charcot. In one of Dr. Perry's patients, an unmarried woman who was gradually drifting into a helpless condition of invalidism, in addition to pallor of the face, the eyes were drawn together and the pinched expression and compressed lips indicated an intensely neurotic condition. This was particularly noticeable during her painful attacks. These were localised in the knee, which was puffy and tender but not reddened. It may be noted that these attacks were not associated with flatulence nor with gastric disturbance, and did not simulate migraine. The day after an attack she would be up, feeling reasonably well, although without appetite and physically depressed. In this case the condition was attributed to the shock of a railway accident, and many of her symptoms were undoubtedly mental. But in this as in other cases the supra-sternal notch was more or less obliterated, and here was to be found the only physical sign that seemed to provide a possible rational indication for treatment. It is here that the interest of the cases largely lies, viz., in the fact that they improved under the use of thyroid extract. As was pointed out in the discussion the administration of thyroid must often be purely empirical. It certainly will not do to restrict its use to those cases alone in which we know, as we know of myxedema, that it will do good. It is only by trial that the extent of its utilities can be discovered, and if by further trial it should be shown that Dr. Perry is correct in attributing to thyroid extract the power of curing the group of symptoms which he has described, we shall have gained a step. In the meantime as an indication for empirical treatment the association of these symptoms with a filling up of the suprasternal notch is a thing to remember.

[1] New York Med. News, Nov. 16.

